# Volatile fragrances associated with flowers mediate host plant alternation of a polyphagous mirid bug

**DOI:** 10.1038/srep14805

**Published:** 2015-10-01

**Authors:** Hongsheng Pan, Yanhui Lu, Chunli Xiu, Huihui Geng, Xiaoming Cai, Xiaoling Sun, Yongjun Zhang, Livy Williams III, Kris A. G. Wyckhuys, Kongming Wu

**Affiliations:** 1 State Key Laboratory for Biology of Plant Diseases and Insect Pests, Institute of Plant Protection, Chinese Academy of Agricultural Sciences, Beijing 100193, China; 2 Instituete of Plant Protection, Xinjiang Academy of Agricultural Sciences, Urumqi 830091, China; 3 Institute of Tea Research, Chinese Academy of Agricultural Sciences, Hangzhou 310008, China; 4 USDA-ARS European Biological Control Laboratory, Campus International de Baillarguet, CS90013 Montferrier sur Lez, St. Gely du Fesc Cedex 34988, France; 5 International Center for Tropical Agriculture CIAT-Asia, Hanoi, Vietnam

## Abstract

*Apolygus lucorum* (Hemiptera: Miridae) is an important insect pest of cotton and fruit trees in China. The adults prefer host plants at the flowering stage, and their populations track flowering plants both spatially and temporally. In this study, we examine whether flower preference of its adults is mediated by plant volatiles, and which volatile compositions play an important role in attracting them. In olfactometer tests with 18 key host species, the adults preferred flowering plants over non-flowering plants of each species. Coupled gas chromatography-electroantennography revealed the presence of seven electrophysiologically active compounds from flowering plants. Although the adults responded to all seven synthetic plant volatiles in electroantennography tests, only four (m-xylene, butyl acrylate, butyl propionate and butyl butyrate) elicited positive behavioral responses in Y-tube olfactometer bioassays. The adults were strongly attracted to these four active volatiles in multi-year laboratory and field trials. Our results suggest that these four fragrant volatiles, which are emitted in greater amounts once plants begin to flower, mediate *A. lucorum*’s preference to flowering host plants. We proved that the use of commonly occurring plant volatiles to recognize a large range of plant species can facilitate host selection and preference of polyphagous insect herbivore.

Within landscapes consisting of mosaics of host and non-host patches, the evolutionary success of herbivorous insects is partly determined by their ability to locate nutritional and oviposition sites in a timely fashion^1^,^2^. For many herbivorous species, host plant selection is mainly mediated through plant volatiles^2^,^3^,^4^,^5^,^6^. To find a host plant, a herbivorous insect must correctly identify the volatiles produced by host plants within a complex background of volatiles produced by non-host plants and the ambient environment^2^,^5^. In general, specialists are more efficient in perceiving host plant cues and finding hosts than generalists^7^. For example, specialist herbivores of solanaceous plants, *Manduca sexta* can locate their host plants by associative olfactory learning^8^,^9^, and analysis of the flowers that are attractive to *M. sexta* adults shows that the scents all have converged on a similar chemical profile^10^. However, to date, the exploitation of host plant volatiles by generalist herbivores does not exhibit any clear tendencies.

The Miridae include several species with extremely broad host plant ranges^11^. For example, *Lygus lineolaris*^12^,^13^, *Lygus hesperus*^14^, and *Lygus rugulipennis*^15^, each utilizes at least 100 host plant species. Host plant phenology appears to mediate, at least in part, between-host dispersal by these mirids so that hosts in the reproductive period are generally favored^11^. Host plant chemical cues play an important role in host recognition, finding, and acceptance by these mirids^16^,^17^,^18^,^19^. Moreover, the role of plant volatiles alone or in combination with insect-derived compounds extends to the next trophic level to guide host finding by mirid natural enemies^20^,^21^. New developments in crop production technology and agronomic practices can change host plant characteristics, and, in turn, alter the pest status of mirids^11^. For example, *Apolygus lucorum* (Meyer-Dür) (Hemiptera: Miridae) was historically considered a minor insect pest of cotton and many other crops in China^22^. However, following the large-scale adoption of transgenic Bt cotton, *A. lucorum* now attain outbreak levels in cotton, and populations spillover to a multitude of other agricultural crops^23^. Recent research has revealed that *A. lucorum* is another polyphagous mirid, with host range exceeding 200 different plant species^24^. It shows a clear preference for plants in reproductive, i.e., flowering stages^25^. Likewise, olfaction is also believed to play a role in the host selection process for *A. lucorum* plant preference^26^. Therefore, we hypothesized that co-occurring plant volatiles emitted at the flowering stage mediate host finding and selection by *A. lucorum*.

In this study, we characterized *A. lucorum* behavioral responses to odors collected from plants at flowering and non-flowering stages for 18 key host plants selected from our previous work^25^. Next, electrophysiological and behavioral responses of *A. lucorum* to plant volatile compounds and individual active compounds were observed in a series of laboratory and field trials. Last, the amounts of electrophysiologically active volatiles were analyzed and compared at flowering and non-flowering stages. This study should help us to understand the host-plant utilization and alternation by *A. lucorum*, and the broader interaction between polyphagous mirid bugs and their host plants.

## Results

### Behavioral responses of *A. lucorum* adults to plants

In Y-tube olfactometer assays of 18 plant species, male and female *A. lucorum* adults preferred odors of flowering plants (FP) versus those of blank controls (CK) or non-flowering plants (NFP) (*P *< 0.05). Comparison of *P* values between genders for each plant species showed that males tended to be more responsive than females (CK vs FP: males = 11, females = 7; NFP vs FP: males = 10, females = 8). Additionally, for both genders, the magnitude of responses (as indicated by *P* values) was usually stronger for trials comparing the blank control versus flowering plants than for trials comparing non-flowering plants versus flowering plants (females: CK vs FP = 12, NFP vs FP = 6; males: CK vs FP = 14, NFP vs FP = 4) (Table 1).

### Electrophysiologically active compounds in flowering host plants

**Figure 1:**
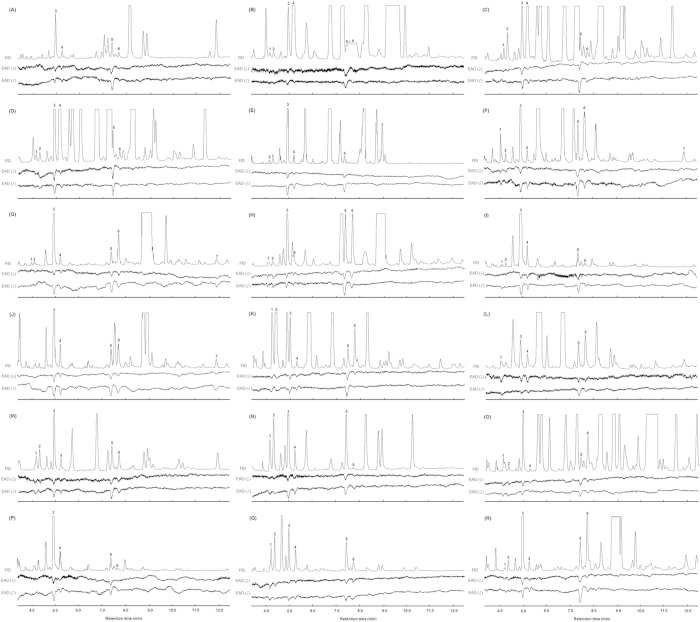
Coupled GC-EADs of adult female and male *Apolygus lucorum* to volatiles from flowering host plants. (**A**) *Agastache rugosus* (Fisch. et Meyer) O. kuntze.; (**B**) *Artemisia annua* L.; (**C**) *Artemisia argyi* Lévl. et Vant.; (**D**) *Artemisia lavandulaefolia* DC.; (**E**) *Artemisia scoparia* Waldst. et Kit.; (**F**) *Cannabis sativa* L.; (**G**) *Chamaemelum nobile* (L.) All.; (**H**) *Chrysanthemum coronarium* L.; (**I**) *Coriandrum sativum* L.; (**J**) *Fagopyrum esculentum* Moench; (**K**) *Gossypium hirsutum* L.; (**L**) *Helianthus annuus* L.; (**M**) *Humulus scandens* (Lour.) Merr.; (**N**) *Impatiens balsamina* L.; (**O**) *Ocimum basilicum* L.; (**P**) *Polygonum orientale* L.; (**Q**) *Ricinus communis* L.; (**R**) *Vigna radiata* (L.) Wilczek. 1: (Z)-3-hexen-1-ol; 2: m-xylene; 3: Butyl acrylate; 4: Butyl propionate; 5: Butyl butyrate; 6: (Z)-3-hexenyl acetate; 7: 3-ethylbenzaldehyde. Five or more replicates were considered to show electrophysiological activity for each plant species.

Coupled GC-EAD revealed, in total, seven electrophysiologically active volatiles from 18 flowering host plants. These volatiles were identified as (Z)-3-hexen-1-ol, m-xylene, butyl acrylate, butyl propionate, butyl butyrate, (Z)-3-hexenyl acetate and 3-ethylbenzaldehyde. At least three of these volatiles were present in all of the plant species tested, and all seven volatiles were present in two of the plant species. Thirteen plant species had six of the EAG-active volatiles. Six of the EAG-active compounds were found in most plant species [(Z)-3-hexen-1-ol, 14 plant species; m-xylene, 14 plant species; (Z)-3-hexenyl acetate, 17 plant species; butyl acrylate, butyl propionate, and butyl butyrate, each in 18 plant species]. 3-ethylbenzaldehyde was recorded from only three plant species (Fig. 1).

### EAG responses of *A. lucorum* adults to synthetic volatiles

**Figure 2:**
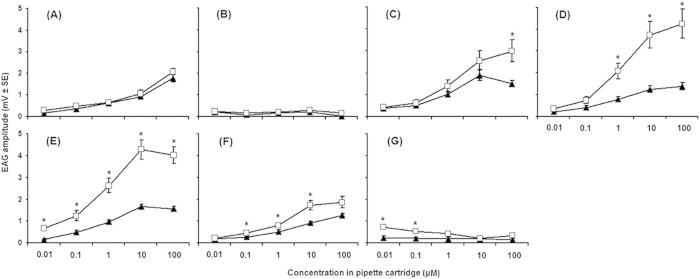
Dose-response curves for EAG responses of female (*filled triangle*) and male (*empty square*) *Apolygus lucorum* antennae to individual compounds identified as the active volatiles of 18 flowering host plants. (**A**) (Z)-3-hexen-1-ol; (**B**) m-xylene; (**C**) Butyl acrylate; (**D**) Butyl propionate; (**E**) Butyl butyrate; (**F**) (Z)-3-hexenyl acetate; (**G**) 3-ethylbenzaldehyde. The data on the y-axis are mean ± SE. *denotes a significant difference between responses of females and males (*P *< 0.05). Ten individuals per gender were assayed for each compound.

Female and male *A. lucorum* adults exhibited electrophysiological responses to seven synthetic volatiles; for five of these volatiles [(Z)-3-hexen-1-ol, butyl acrylate, butyl propionate, butyl butyrate, and (Z)-3-hexenyl acetate], the EAG values generally increased as concentration increased. For some concentrations, EAG responses of males to butyl acrylate, butyl propionate, butyl butyrate, (Z)-3-hexenyl acetate and 3-ethylbenzaldehyde were significantly greater than those of females (*P *< 0.05) (Fig. 2).

### Behavioral responses of *A. lucorum* adults to synthetic volatiles

**Figure 3:**
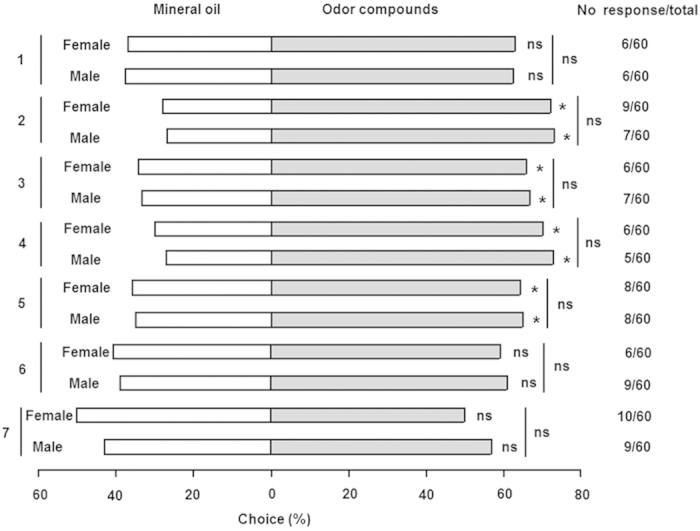
Behavioral responses of adult female and male *Apolygus lucorum* to individual compounds in Y-tube olfactometer assays. 1: (Z)-3-hexen-1-ol; 2: m-xylene; 3: Butyl acrylate; 4: Butyl propionate; 5: Butyl butyrate; 6: (Z)-3-hexenyl acetate; 7: 3-ethylbenzaldehyde. *P < 0.05; ns, not significant. Sixty individuals per gender were tested for each compound.

In Y-tube olfactometer assays, four of seven electrophysiologically active volatiles elicited attraction among *A. lucorum* adults. Adults showed significant movement upwind to m-xylene (female, *P *= 0.036; male, *P *= 0.039), butyl acrylate (female, *P *= 0.003; male, *P *= 0.010), butyl propionate (female, *P *= 0.014; male, *P *= 0.022) and butyl butyrate (female, *P *= 0.001; male, *P *= 0.002). There was no difference in response between genders for any of the seven EAG-active compounds (*P *> 0.05) (Fig. 3).

### Greenhouse evaluation of different plant volatiles attractive to adult *A. lucorum*

**Figure 4:**
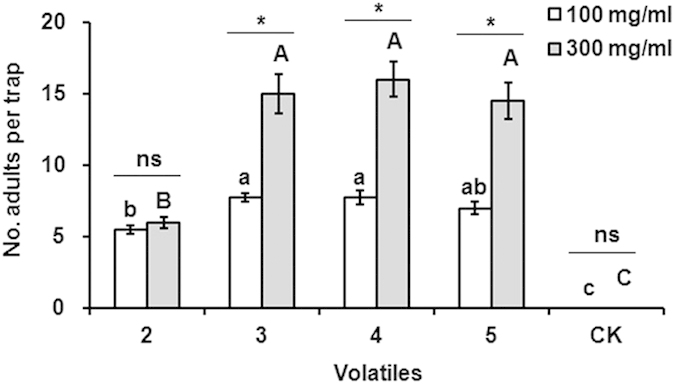
Captures of adult *Apolygus lucorum* in bucket traps in greenhouse cage experiments. Before each trial, 200 *A. lucorum* adults were released into each cage. 2: m-xylene; 3: Butyl acrylate; 4: Butyl propionate; 5: Butyl butyrate. The data on the y-axis are mean ± SE. For each release concentration, significant differences (*P *< 0.05) between compounds are denoted by different letters (uppercase letters, 300 mg/ml; lowercase letters, 100 mg/ml). *denotes a significant difference between two doses (*P *< 0.05), and ns shows no difference (*P *> 0.05). Four replicates were conducted for each dose.

For the four active synthetic volatiles identified in the behavioral study above (m-xylene, butyl acrylate, butyl propionate and butyl butyrate), the number of trapped bugs was significantly higher than that of the control in each trial (100 mg/ml: *P *< 0.001; 300 mg/ml: *P *< 0.001). The sex ratio (female/male) of *A. lucorum* adults trapped by these volatiles was 1:1.55, and no significant difference was found between sex ratios of tested individuals (1:1) and trapped individuals (*P *= 0.107), and between those trapped by different volatiles (*P *= 0.847). At 100 mg/ml, trap catches for butyl acrylate and butyl propionate were not different from each other, but were each greater than m-xylene. Catches for butyl butyrate were not different from m-xylene, butyl acrylate or butyl propionate. At 300 mg/ml, trap catches for the three esters, butyl acrylate, butyl propionate, and butyl butyrate, were not different from each other, but were significantly greater than for m-xylene. Furthermore, significantly more *A. lucorum* adults were trapped with 300 mg/ml volatiles than with 100 mg/ml (butyl acrylate: *P *= 0.002, butyl propionate: *P *< 0.001, and butyl butyrate: *P *= 0.001), but not for m-xylene (*P *= 0.356) (Fig. 4).

### Field evaluation of different plant volatiles attractive to adult *A. lucorum*

**Figure 5:**
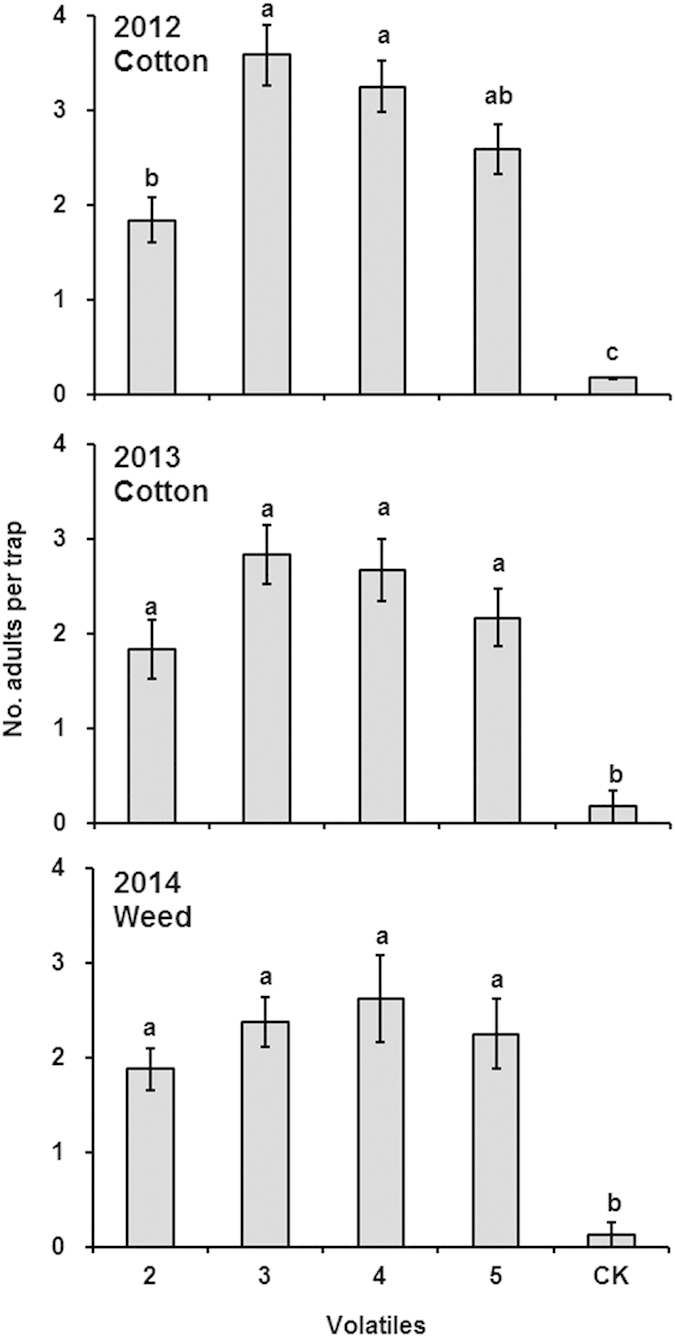
Captures of *Apolygus lucorum* adults in the field during 2012–2014. Volatile 2: m-xylene; 3: Butyl acrylate; 4: Butyl propionate; 5: Butyl butyrate. 2012 and 2013: cotton fields with twelve and six replicates, respectively; 2014: weed (mainly *Artemisia argyi*) field with eight replicates. The data on the y-axis are mean ± SE. Different letters denote significant differences between treatments (*P *< 0.05).

Significantly more *A. lucorum* adults were captured in bucket traps with each of the four plant-derived volatiles (m-xylene, butyl acrylate, butyl propionate and butyl butyrate) than with the control from 2012 to 2014 (*P *< 0.001 each year). In 2012, captures from butyl acrylate and butyl propionate traps were significantly greater than for m-xylene traps; but in 2013 and 2014, no significant difference was found between the four active volatiles, although trends were similar to those for 2012. The sex ratios (female/male) of adults trapped by four active volatiles were 1:4.89, 1:2.35, 1:1.43, and those in the fields were 1:2.69, 1:1.82, 1:1.19 in 2012, 2013, 2014, respectively. No significant difference in sex ratio was found between field and trapped individuals (*P *> 0.05), and between those trapped by different plant-derived volatiles (*P *> 0.05) (Fig. 5).

### Comparison of the amounts of the four active volatiles in flowering and non-flowering plants

**Figure 6:**
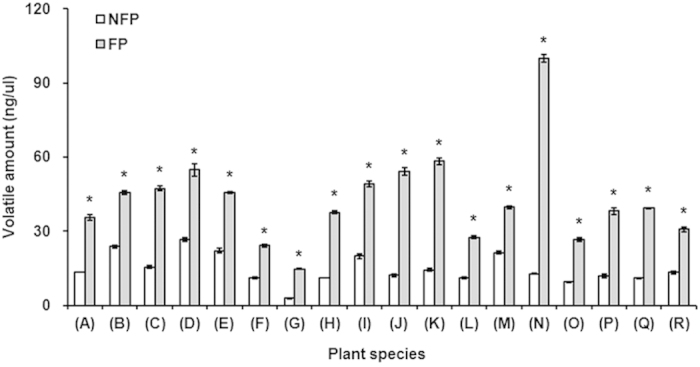
The total amount of four active volatiles in flowering and non-flowering host-plant species. 2: m-xylene; 3: Butyl acrylate; 4: Butyl propionate; 5: Butyl butyrate. (**A**) *Agastache rugosus* (Fisch. et Meyer) O. kuntze.; (**B**) *Artemisia annua* L.; (**C**) *Artemisia argyi* Lévl. et Vant.; (**D**) *Artemisia lavandulaefolia* DC.; (**E**) *Artemisia scoparia* Waldst. et Kit.; (**F**) *Cannabis sativa* L.; (**G**) *Chamaemelum nobile* (L.) All.; (**H**) *Chrysanthemum coronarium* L.; (**I**) *Coriandrum sativum* L.; (**J**) *Fagopyrum esculentum* Moench; (**K**) *Gossypium hirsutum* L.; (**L**) *Helianthus annuus* L.; (**M**): *Humulus scandens* (Lour.) Merr.; (**N**): *Impatiens balsamina* L.; (**O**) *Ocimum basilicum* L.; (**P**) *Polygonum orientale* L.; (**Q**) *Ricinus communis* L.; (**R**) *Vigna radiata* (L.) Wilczek. NFP: Non-flowering (vegetative) plants; FP: Flowering plants. *denotes a significant difference within the same plant species between flowering and non-flowering stages (*P *< 0.05). A total of six volatile samples were analyzed at each growth stage of each plant.

For all 18 tested host plants, the amount of each of the four active volatiles (i.e., m-xylene, butyl acrylate, butyl propionate and butyl butyrate) generally increased after entering the flowering stage, and the total quantity of these four active volatiles in each plant species was significantly (1.86–7.82-fold) higher when flowers were present (*P *< 0.001 for all) (Fig. 6, see Supplementary Table S1 online).

## Discussion

Results of this study are consistent with previous work that indicates that *A. lucorum* shows a marked preference for host plants at the flowering stage, and that host plant phenology plays an important role in seasonal dispersal between hosts by *A. lucorum* in agricultural landscapes^23^,^25^,^27^. Dong *et al*.^26^ noted that *A. lucorum* adults and nymphs had higher survival rates and population fitness when on flowering plant species compared to related non-flowering plants. This positive relationship between adult flower preference and offspring performance further highlights the significance of flowers in seasonal dynamics and populations of the generalist *A. lucorum*. This phenomenon has also been recorded for other generalist herbivores, such as *Lygus* bugs^11^,^28^ and lepidopteran insects^29^. Hence, exploring the mechanisms underlying the flower preferences of these generalist herbivores may help researchers to fully understand the population dynamics, interplant movements and the interactions between generalist herbivores and host plants.

Previous studies with other mirid bugs suggest gender-specific responses to volatiles of different chemical classes. Specifically, electrophysiological and behavioral studies suggest that male mirid bugs are usually more sensitive to acetates, either insect- or plant-produced, than female individuals, and that females are more responsive to plant-produced compounds in other classes^16^,^18^,^30^,^31^,^32^,^33^. In the present study, we found that both sexes were responsive to host plant odors but male *A. lucorum* antennae were more responsive than female antennae to esters, and the electrophysiologically active compounds elicited a behavioural response involving locomotion towards these compounds (butyl acrylate, butyl propionate and butyl butyrate) in the laboratory, greenhouse, and field trials. Our results are consistent with the hypothesis that male mirid bugs are generally more responsive to acetates, while female bugs are more sensitive to other compounds. However, this hypothesis is based on studies of relatively few mirid bug species; thus, additional work on other species is warranted.

Although each plant species shows a unique volatile profile, there is often considerable overlap between species. For example, *M. sexta* visited flowers, although they differ qualitatively and quantitatively in their scent profiles, emit characteristic compounds, such as methyl benzoate, benzyl alcohol, and benzaldhyde^10^. In this study, each plant species showed a large difference in size and architecture; however, they all emitted at least four volatiles that elicited EAG and positive behavioral responses in adult *A. lucorum*. These four volatiles were present in significantly greater levels in each plant species during the flowering period compared to non-flowering periods. These data indicated that the increased level of these four volatiles was the major reason underlying the marked preference of *A. lucorum* adults for flowering host plants. From an evolutionary perspective, the use of commonly occurring plant volatiles to recognize a large range of plant species may also facilitate learning behavior and preference for the host that is more abundant in local agricultural landscapes. The finding supported our hypothesis but was not consistent with the common viewpoint that generalist insects typically utilize many different plant-derived volatiles to find different host plants^4^.

In this study, we conducted a series of experiments on the electrophysiological and behavioral responses of *A. lucorum* to plant volatiles in order to better understand host recognition and selection by the mirid bug. Our results demonstrate that *A. lucorum* perceives and orients toward several plant-derived volatiles that are emitted at higher levels when plants are in the flowering stage, and that this relationship plays an important role in host-plant alternation by the mirid bug. Our use of several types of behavioral experiments was a matter of necessity because of the great variability that is sometimes observed between different types of arenas, as well as laboratory and field settings. In particular, our data set from the Y-tube olfactometer study is a good example of why it is valuable to include odors for all choices in an olfactometer assay, i.e., “something vs something” as opposed to an odor compared to a clean air control, i.e., “something vs nothing”, as well as following-up with field studies to verify (or refute) the results found in the laboratory^34^. As would be expected, our Y-tube data showed stronger significance, as measured by *P* values, for the clean air vs flower odor tests, “something vs nothing”, than for the vegetative odor vs flower odor tests, “something vs something”. The nearly 3-fold difference that we observed reflected the fact that “something vs something” tests require the insect to make a more discerning choice than tests of an odor vs clean air. Reliance solely on the tests of clean air vs flower odors would have presented a more liberal perspective of our results. Thus, behavioral studies may require a variety of approaches that use different strategies in the laboratory (e.g., Y-tube olfactometer) and field to understand the effect of volatile compounds on insect behavior^35^. In particular, in the complex field environment that is characterized by a multitude of potential cues, volatile and otherwise, use of traps baited with volatile compounds may reveal important information on the actual host-plant preferences of phytophagous insects. This powerful assessment tool can also be applied to the further development of attractants of insect pests, which are widely used in pest behavioral manipulation.

Our improved knowledge of *A. lucorum*-plant volatile interactions can be incorporated into existing management strategies. For example, trap crop (mungbean) and a repellent compound (dimethyl disulfide) have been successfully selected and applied in the management of *A. lucorum* 36,37. These studies indicated that behavioral manipulation is a viable option for preventing the damage of *A. lucorum*. In the present study, the four bio-active plant volatiles that were identified provide a basis for further development of attractants for *A. lucorum*. Additionally, the combination of attractant and trap crop for enhancing attractiveness^38^, and attractant and repellent for developing push-pull strategy^39^,^40^ must be further assessed. These alternative environmentally-friendly measures are needed for controlling *A. lucorum*^41^.

Our study provides chemistry-based insights into flower preference by *A. lucorum* adults, which supplies basic information for further developing behavioral manipulation measures for control. It also lays a solid foundation for exploring a new pattern of host selection and volatile identification of generalist herbivores, which is available for fully understanding the interaction and co-evolution between polyphagous herbivores and their host plants.

## Methods

### Insects and plants

Adults and nymphs of *A. lucorum* were collected between July and August each year from cotton fields at the Langfang Experiment Station of the Chinese Academy of Agricultural Sciences (CAAS, 39.53 °N, 116.70 °E) (Hebei Province, China). The colonies were maintained on green bean pods (*Phaseolus vulgaris*) and 10% sucrose solution^42^. For all the behavioral and electrophysiological trials, 6- to 8-d old unmated female and male *A. lucorum* adults were used. Adults were collected within 24 h of emergence, separated by gender and maintained in different rearing containers to prevent mating.

Based on our previous work^25^, 18 of the most important host plants which normally support abundant *A. lucorum* populations were chosen (see Supplementary Table S2 online). All plants were planted on two separate occasions (early May and late June) by direct seeding or transplanting into pots at the Langfang Experiment Station of CAAS. Plots were managed using identical agronomic practices, and no insecticides were used^23^. All plants were caged (80 mesh) to prevent damage by herbivorous insects and mites. Vegetative and flowering periods of each plant species were selected for this study. We sampled plants at the vegetative growth stage ca. 2 weeks before the first emergence of flower buds, and the flowering growth stage was sampled at the time of peak bloom.

### Behavioral responses of *A. lucorum* adults to different plants

Insect behavioral responses to plant odors are commonly analyzed using a Y-tube olfactometer^43^. The olfactometer had the following specifications: a 3-cm inner diameter clear glass tube with a 15-cm-long central tube and two 15-cm-long lateral arms with a 60° angle at the Y-junction. The apparatus was placed in a 100 × 100 × 60-cm chamber that was illuminated with two 40-W fluorescent lamps (light intensity 2000 lux), maintained at 25 ± 1 °C, 60 ± 5% RH. A vacuum pressure pump (Beijing Institute of Labor Instrument, Beijing, China) pushed air through activated charcoal and an Erlenmeyer flask filled with distilled water. Airflow through each of the olfactometer arms was maintained at 300 ml/min and entered the apparatus via Teflon tubing. When a given test plant was in full bloom, the branches containing the flowers were removed with scissors and immediately introduced into one holding chamber for the olfactometer assay. In a second chamber, we introduced the branches cut from the same plant species at the vegetative period. The branches of some plant species were large enough so that only one was used for each assay; branches of other species were small, which allowed several to be used together. Due to great variability in size and architecture among plant species, the total amount of plant tissue was not standardized between all plant species; instead the same weight of plant tissue was used in the two chambers for each trial. The excised ends of the branches were wrapped with moist cotton towels and enclosed inside a plastic bag before they were placed in the holding chambers. Prior to behavioral bioassays, insects were starved for 4 h. Each insect was used only once. Plant tissue was replaced every hour, and the Y-tube olfactometer was replaced with a clean one after four individuals had been tested. For each treatment, we tested 60 insects per gender. All bioassays were conducted between 08:00 h and 18:00 h.

### Collection of plant volatiles

Headspace sampling was used to collect the volatiles from different plant species^44^. More specifically, once a plant reached the vegetative period or flowering period, pots were moved to the laboratory for volatile collection. The plant material samples were the same as those used in the above olfactometer assay. The selected plant parts were sealed in a polyester cooking bag (unprinted 45 × 55-cm bags, Terinex, Bedford, UK). Clean air was introduced from the lower part of the bag, and allowed out of the top through a volatile collection trap, i.e., a plastic tube containing 40 mg of 80–100 mesh Porapak Type Q adsorbent (Bulk Packing Material, Altech Assoc. USA). The collection of volatiles was replicated six times for a 4 h time period (16:00–20:00 h), which coincides with the period of the greatest activity of *A. lucorum* adults in the field^45^. Volatiles were eluted with 400 μl HPLC-grade dichloromethane (Fisher, Fair Lawn, NJ). The solutions were stored at −20 °C in 1.5 ml glass vials (Agilent, USA).

### Coupled gas chromatography-electroantennography detection (GC-EAD)

A coupled GC-EAD system was used, in which the effluent from the GC column was simultaneously directed to both the antennal preparation and the GC detector^44^. The heads of female or male *A. lucorum* adult were excised using a scalpel, and tips of the antennae were removed to ensure good contact with the electrode. The reference electrode was inserted into the head cavity, and the recording electrode was attached to both antennae. The reference and recording electrodes were silver wires enclosed in drawn glass capillary tubes filled with phosphate buffered saline^18^. Ten coupled runs were completed for each gender × plant species × plant growth stage combination. Only flame ionization detector (FID) peaks that corresponded to an electroantennography (EAG) peak in five or more replicates were considered to show electrophysiological activity.

### Identification of electrophysiologically active volatiles

Identification of FID peaks showing electrophysiological activity was performed by positive ion electron impact gas chromatography-mass spectrometry (EI GC-MS) on an HP 6890 GC coupled to an HP 5973 MS detector. One microliter samples were injected at 250 °C onto the HP-5MS column. These volatiles were tentatively identified by comparing mass spectra with those of authentic samples in the NIST 2005 database and were further confirmed by co-injecting the collection of plant volatiles with commercially available authentic standards on both non-polar HP-5MS and polar DB-WAX columns (30 m × 0.25 mm × 0.25 μm, Agilent Technologies, Palo Alto, CA, USA), with peak enhancement indicating co-elution. Quantification of identified compounds was achieved by injecting authentic standards at four known concentrations onto the HP-5MS column and recording the peak area. Each standard was injected four times, and a calibration curve was plotted for each compound, which was used to determine the amounts of each active volatile from the 18 tested plant species during both flowering and non-flowering (=vegetative) periods. To compare the amounts of each active volatile, six samples were analyzed at flowering and non-flowering stages of each plant species, respectively.

### Electroantennography

The EAG apparatus (Syntech Ltd., Hilversum, The Netherlands) was linked to a desktop computer with an IDAC-02 data acquisition interface board, on which EAG responses were recorded, stored and quantified. Electrical signals from the antennae were amplified using a high-impedance DC amplifier and a signal connection interface box (Auto Spike, IDAC2/3; Syntech). The antennae of female and male *A. lucorum* adults were prepared as above, and were used as detectors to identify potential activity of given volatiles. The reference and recording electrodes were prepared according to the GC-EAD methods described above. Seven authentic chemical standards were used for EAG: (Z)-3-hexen-1-ol (98%), m-xylene (99%), butyl acrylate (99%), butyl propionate (99%), butyl butyrate (98%), (Z)-3-hexenyl acetate (97%) and 3-ethylbenzaldehyde (98%) (Sigma Aldrich, Gillingham, UK).

For the EAG trials, serial dilutions (0.01, 0.1, 1.0, 10 and 100 μM) of each chemical were made using paraffin oil (Sigma Aldrich, UK). Stimulus applicators were prepared by pipetting 10 μl of the test dilution onto a 5 cm × 0.5 cm strip of filter paper, after which the filter paper was placed inside a 15-cm-long glass Pasteur pipette. Four controls were used: 1) an empty pipette; 2) a pipette containing only a strip of filter paper; 3) a pipette containing 10 μl of paraffin oil on filter paper; and 4) a pipette containing 10 μl of standard (100 μg/μl paraffin oil-dissolved nonanal, 97%, Sigma Aldrich, UK) on filter paper.

EAG recording began 3 min after the antennal preparation and used the following test protocol. The controls were tested in the following order (1, 2, 3, 4), after which low-to-high serial dilutions (0.01, 0.1, 1.0, 10 and 100 μM) of the given chemical were tested, then the controls 4 and 3. Presentation of controls throughout the recording session permitted standardization of antennal responses and correction for antennal fatigue. Tested chemicals and controls were applied at 0.5-sec pulses, at 30-sec intervals separated by purges of filtered, humidified air. Each antennal preparation only tested one chemical with five dilutions. EAGs were measured as the maximum amplitude of depolarization (mV) and were analyzed using a customized software package (Syntech). For each chemical, 10 *A. lucorum* adults of each gender were tested.

### Behavioral responses of *A. lucorum* adults to synthetic volatiles

The Y-tube olfactometer was used to test behavioral responses of *A. lucorum* adults to each of the seven synthetic volatiles. The protocols and methods, as well as the setup of the olfactometer, are described as above. During behavioral trials, a single chemical was diluted in amounts equivalent to those used in the EAG assays. We applied 10 μl (1 μg/μl) of the dilution onto a 5 cm × 0.5 cm piece of filter paper and placed the paper into a flask (10 ml, Beijing Kenin Industrial Biotechnology Co. LTD, Beijing, China). In these experiments, female and male *A. lucorum* adults were offered a choice between a given chemical and mineral oil, which was used as solvent for the chemical. New filter papers were used for each insect. For each treatment, we tested 60 replicates per gender.

### Greenhouse evaluation of *A. lucorum* attraction

Cage experiments were conducted to determine the level of attractiveness of four active volatiles (i.e., m-xylene, butyl acrylate, butyl propionate and butyl butyrate) selected from the Y-tube olfactometer assays to *A. lucorum* adults in the greenhouse. These compounds were chosen based on their activity during the Y-tube assays. During the experiments, two pots of cotton plants (20–25-cm height; 8–10 leaves) were placed in the center of a 2 × 2 × 2-m screen cage (80 mesh). A cotton wick soaked in 5% honey water was placed at the center of the cage for feeding. A bucket trap (Pherobio Technology Co., LTD., China; 15 cm in diameter, 12.5 cm in height) was placed in each corner of the cage, and another one was placed in the center of the cage. All five traps were hung 10–15 cm above the plant tops, and four randomly-selected traps were baited with synthetic volatiles (one volatile per trap), with the remaining trap containing the solvent-only control. Synthetic volatiles dissolved in the solvent lanolin or the single solvent were placed in open 1.5-ml glass vials, and fixed to the middle of the trap. Next, 200 *A. lucorum* adults (1:1 sex ratio) were released into the cage. At 3 d post-release, we counted all trapped individuals and determined their gender. Two experiments using different volatile rates were conducted, (A) 1 ml of 100 mg/ml volatile per trap; and (B) 1 ml of 300 mg/ml volatile per trap. Each set of experiments was repeated four times. Experiments were conducted at 25 ± 1 °C and 60 ± 5% RH.

### Field evaluation of *A. lucorum* attraction

Field trials were performed to evaluate the behavioral response of *A. lucorum* adults to four active volatiles (m-xylene, butyl acrylate, butyl propionate and butyl butyrate) at the Langfang Experimental Station of the CAAS, during 2012–2014. Bucket traps were placed in a cotton (*Gossypium hirsutum*) field in August of 2012–2013 and in a weed (mainly *Artemisia argyi*) field in early-September of 2014, at a height of 1.8 m above the ground (i.e., ca. 20 cm above at the plant canopy level). At this height, *A. lucorum* was found to most abundant and active^45^. Within the field, traps were spaced at least 15 m apart, arranged randomly and oriented uniformly in an east-west fashion in the field. Synthetic volatiles dissolved in the solvent lanolin (1 ml, 100 μg/μl) or the single solvent (i.e., as a control) were placed in open 1.5-ml glass vials, and stuck to the middle of sticky card. Traps were collected 3 d later, and trapped *A. lucorum* adults of each gender were recorded. Each volatile treatment was replicated 12, 6 and 8 times (one trap for each replicate) in 2012, 2013 and 2014, respectively.

### Statistical analyses

For Y-tube olfactometer bioassays, the null hypothesis that *A. lucorum* showed no preference for either olfactometer arm (i.e., 50:50 response) was analyzed using χ^2^ goodness-of-fit test. The individuals that remained unresponsive were not included in the analysis. EAG amplitudes (mean ± SE) were control-adjusted and presented as responses relative to the standard, which was 100 μM nonanal. Trap data were log-transformed (n + 1) to fit the assumption of homogeneity of variance for analysis of variance (ANOVA). Means were compared using ANOVAs followed by Tukey’s honestly significant differences (HSD) test, whereas the Kruskal-Wallis test was conducted on data sets that were not normally distributed. The difference in sex ratio between trapped and tested individuals in greenhouse evaluation and between trapped and field-collected individuals in the field trial was analyzed using χ^2^ goodness-of-fit test. Differences in volatile amounts between flowering and non-flowering plants were determined using analyses of variance (ANOVA) followed by Tukey’s HSD test. SAS statistical software package was used for data analysis^46^.

## Additional Information

**How to cite this article**: Pan, H. *et al*. Volatile fragrances associated with flowers mediate host plant alternation of a polyphagous mirid bug. *Sci. Rep*. **5**, 14805; doi: 10.1038/srep14805 (2015).

## Supplementary Material

Supplementary Information

## Figures and Tables

**Table 1:** Behavioral responses of female and male adult *Apolygus lucorum* to flowering and non-flowering plants in Y-tube olfactometer tests.

Plant species	CK vs. FP		NFP vs. FP	
	Female	Male	Female	Male
*Agastache rugosus* (Fisch. et Meyer) O. kuntze.	14/35 (0.003)	14/37 (0.001)	17/34 (0.017)	14/35 (0.003)
*Artemisia annua* L.	18/36 (0.014)	18/34 (0.027)	14/37 (0.001)	19/35 (0.029)
*Artemisia argyi* Lévl. et Vant.	16/34 (0.011)	13/39 (<0.001)	15/34 (0.007)	15/40 (<0.001)
*Artemisia lavandulaefolia* DC.	18/38 (0.008)	16/37 (0.004)	16/36 (0.006)	18/37 (0.010)
*Artemisia scoparia* Waldst. et Kit.	16/35 (0.008)	17/38 (0.005)	16/32 (0.021)	16/31 (0.029)
*Cannabis sativa* L.	18/37 (0.010)	17/34 (0.017)	17/31 (0.043)	18/33 (0.036)
*Chamaemelum nobile* (L.) All.	19/36 (0.022)	19/35 (0.029)	17/33 (0.024)	16/32 (0.021)
*Chrysanthemum coronarium* L.	18/37 (0.010)	16/36 (0.006)	15/38 (0.002)	17/34 (0.017)
*Coriandrum sativum* L.	16/34 (0.011)	18/33 (0.036)	16/32 (0.021)	17/31 (0.043)
*Fagopyrum esculentum* Moench	17/35 (0.013)	18/33 (0.036)	19/34 (0.039)	20/36 (0.033)
*Gossypium hirsutum* L.	17/34 (0.017)	18/37 (0.010)	18/33 (0.036)	19/34 (0.039)
*Helianthus annuus* L.	18/37 (0.010)	19/35 (0.029)	19/38 (0.012)	18/37 (0.010)
*Humulus scandens* (Lour.) Merr.	16/36 (0.006)	15/35 (0.005)	17/32 (0.032)	18/33 (0.036)
*Impatiens balsamina* L.	18/34 (0.027)	12/37 (< 0.001)	17/32 (0.032)	17/33 (0.024)
*Ocimum basilicum* L.	19/37 (0.016)	18/39 (0.005)	16/38 (0.003)	18/38 (0.008)
*Polygonum orientale* L.	19/35 (0.029)	16/34 (0.011)	17/33 (0.024)	16/33 (0.015)
*Ricinus communis* L.	15/38 (0.002)	17/37 (0.006)	17/33 (0.024)	15/32 (0.013)
*Vigna radiata* (L.) Wilczek	17/36 (0.009)	15/37 (0.002)	18/32 (0.048)	17/34 (0.017)

CK: Blank control; NFP: Non-flowering (vegetative) plants; FP: Flowering plants. Numbers in front and after backslashes (/) denote the number of adults preferring CK and FP or NFP and FP, respectively. Of the 60 individuals tested per treatment, the number of no-responses ranged from 3 to 13. Data in brackets show *P* values from χ^2^ goodness-of-fit test.
