# Supplementary material for: Volatile fragrances associated with flowers mediate host plant alternation of a polyphagous mirid bug
Source: Sci Rep. 2015 Oct 1;5:14805. doi: 10.1038/srep14805 (PMC4589772; doi:10.1038/srep14805)
Supplement: Supplementary Information [file srep14805-s1.pdf]

Supplementary Information

Volatile fragrances associated with flowers mediate host plant alternation of a polyphagous mirid bug

Hongsheng Pan[1,2], Yanhui Lu[1], Chunli Xiu[1], Huihui Geng[1], Xiaoming Cai[3], Xiaoling Sun[3], Yongjun Zhang[1], Livy Williams III[4], Kris A. G. Wyckhuys[5] & Kongming Wu[1]

[1]State Key Laboratory for Biology of Plant Diseases and Insect Pests, Institute of Plant Protection, Chinese Academy of Agricultural Sciences, Beijing 100193, China, [2]Instituete of Plant Protection, Xinjiang Academy of Agricultural Sciences, Urumqi 830091, China, [3]Institute of Tea Research, Chinese Academy of Agricultural Sciences, Hangzhou 310008, China, [4]USDA-ARS European Biological Control Laboratory, Campus International de Baillarguet, CS90013 Montferrier sur Lez, St. Gely du Fesc Cedex 34988, France, [5]International Center for Tropical Agriculture CIAT-Asia, Hanoi, Vietnam.

** Correspondence author: yhlu@ippcaas.cn*

Table S1. Quantification of the four active volatiles in flowering and non-flowering host-plant species. 2: m-xylene; 3: Butyl acrylate; 4: Butyl propionate; 5: Butyl butyrate. NFP: Non-flowering (vegetative) plants; FP: Flowering plants. Means (± SE) followed by different letters within the same plant species denote significant differences between flowering and non-flowering stages ($P < 0.05$). A total of six volatile samples were analyzed at each growth stage of each plant.

Plant species	Growth stage	The amount of active volatiles (ng/μl)			
		2	3	4	5
Agastache rugosus (Fisch. et Meyer) O. kuntze.	NFP	3.72 ± 0.10 a	4.43 ± 0.06 b	1.00 ± 0.05 b	4.30 ± 0.17 b
	FP	0.59 ± 0.02 b	24.53 ± 0.91 a	2.76 ± 0.17 a	7.65 ± 0.44 a
Artemisia annua L.	NFP	1.25 ± 0.04 b	15.36 ± 0.38 b	1.57 ± 0.09 b	5.62 ± 0.30 a
	FP	1.85 ± 0.10 a	31.46 ± 1.13 a	6.03 ± 0.20 a	6.13 ± 0.23 a
Artemisia argyi Lévl. et Vant.	NFP	1.73 ± 0.07 b	11.10 ± 0.39 b	1.20 ± 0.06 b	1.49 ± 0.12 b
	FP	5.83 ± 0.49 a	28.01 ± 0.99 a	6.35 ± 0.19 a	7.07 ± 0.67 a
Artemisia lavandulaefolia DC.	NFP	0.89 ± 0.04 b	19.12 ± 0.62 b	2.25 ± 0.08 b	4.29 ± 0.07 b
	FP	1.80 ± 0.06 a	39.29 ± 2.39 a	4.54 ± 0.08 a	9.12 ± 0.36 a
Artemisia scoparia Waldst. et Kit.	NFP	0.08 ± 0.00 b	20.70 ± 0.96 b	0.79 ± 0.09 b	0.52 ± 0.09 b
	FP	0.15 ± 0.00 a	40.85 ± 0.41 a	2.87 ± 0.01 a	1.60 ± 0.02 a
Cannabis sativa L.	NFP	0.03 ± 0.00 b	9.56 ± 0.57 b	1.10 ± 0.04 b	0.41 ± 0.01 b
	FP	0.20 ± 0.01 a	20.62 ± 0.54 a	2.05 ± 0.04 a	1.20 ± 0.07 a
Chamaemelum nobile (L.) All.	NFP	0.04 ± 0.00 b	1.48 ± 0.08 b	0.11 ± 0.01 b	1.13 ± 0.07 b
	FP	0.29 ± 0.01 a	10.85 ± 0.33 a	0.90 ± 0.03 a	2.63 ± 0.10 a
Chrysanthemum coronarium L.	NFP	0.43 ± 0.02 b	5.02 ± 0.29 b	1.37 ± 0.04 b	4.20 ± 0.15 b
	FP	0.66 ± 0.01 a	21.11 ± 1.09 a	3.03 ± 0.29 a	12.84 ± 0.89 a
Coriandrum sativum L.	NFP	0.02 ± 0.00 b	18.47 ± 1.03 b	1.22 ± 0.08 b	0.23 ± 0.01 b
	FP	0.09 ± 0.00 a	44.76 ± 1.30 a	2.24 ± 0.07 a	1.95 ± 0.06 a
Fagopyrum esculentum Moench	NFP	0.14 ± 0.00 b	8.97 ± 0.57 b	0.84 ± 0.06 b	2.11 ± 0.08 b
	FP	0.40 ± 0.02 a	43.70 ± 1.67 a	2.73 ± 0.08 a	7.40 ± 0.32 a
Gossypium hirsutum L.	NFP	2.67 ± 0.15 b	4.65 ± 0.47 b	2.47 ± 0.22 b	4.52 ± 0.27 b
	FP	4.09 ± 0.35 a	39.03 ± 1.61 a	3.81 ± 0.13 a	11.32 ± 0.95 a
Helianthus annuus L.	NFP	1.87 ± 0.04 a	2.07 ± 0.04 b	0.78 ± 0.04 b	6.39 ± 0.34 b
	FP	1.60 ± 0.06 b	12.11 ± 0.40 a	5.10 ± 0.15 a	8.68 ± 0.24 a
Humulus scandens (Lour.) Merr.	NFP	10.11 ± 0.48 a	4.13 ± 0.24 b	1.43 ± 0.10 b	5.67 ± 0.03 b
	FP	5.21 ± 0.33 b	19.88 ± 0.68 a	3.71 ± 0.15 a	10.84 ± 0.42 a
Impatiens balsamina L.	NFP	4.12 ± 0.09 b	3.32 ± 0.06 b	1.29 ± 0.06 b	4.05 ± 0.06 b
	FP	21.97 ± 0.14 a	51.56 ± 1.34 a	6.81 ± 0.10 a	19.53 ± 0.16 a
Ocimum basilicum L.	NFP	1.29 ± 0.02 b	3.05 ± 0.09 b	0.94 ± 0.04 b	4.17 ± 0.11 b
	FP	4.87 ± 0.14 a	9.65 ± 0.41 a	4.06 ± 0.20 a	7.93 ± 0.23 a
Polygonum orientale L.	NFP	0.03 ± 0.00 b	10.74 ± 0.76 b	0.54 ± 0.04 b	0.55 ± 0.02 b
	FP	0.15 ± 0.01 a	33.42 ± 1.31 a	2.82 ± 0.10 a	1.79 ± 0.13 a
Ricinus communis L.	NFP	0.16 ± 0.01 b	2.07 ± 0.03 b	0.97 ± 0.04 b	7.74 ± 0.22 b
	FP	10.15 ± 0.28 a	13.88 ± 0.23 a	6.18 ± 0.07 a	9.07 ± 0.25 a
Vigna radiata (L.) Wilczek	NFP	1.79 ± 0.10 a	1.56 ± 0.09 b	0.72 ± 0.04 b	9.19 ± 0.65 a
	FP	1.31 ± 0.07 b	15.48 ± 1.35 a	4.48 ± 0.24 a	9.51 ± 1.06 a

Table S2. List of *Apolygus lucorum* host plants included in the study

Scientific name	Common name	Variety	Source
Agastache rugosus (Fisch. et Meyer) O. kuntze.	Wrinkled gianthyssop herb		Bred by Institute of Vegetables and Flowers (IVF), Chinese Academy of Agricultural Sciences (CAAS) (Beijing, China)
Artemisia annua L.			Transplanted from agricultural fields
Artemisia argyi Lévl. et Vant.			Transplanted from agricultural fields
Artemisia lavandulaefolia DC.			Transplanted from agricultural fields
Artemisia scoparia Waldst. et Kit.			Transplanted from agricultural fields
Cannabis sativa L.	Hemp		Obtained from IVF, CAAS
Chamaemelum nobile (L.) All.	Chamomile		Obtained from IVF, CAAS
Chrysanthemum coronarium L.	Crowndaisy		Obtained from IVF, CAAS
Coriandrum sativum L.	Caraway		Obtained from IVF, CAAS
Fagopyrum esculentum Moench	Buckwheat		Obtained from IVF, CAAS
Gossypium hirsutum L.	Cotton	GK12	Bred by Biotechnology Research Institute, CAAS (Beijing, China)
Helianthus annuus L.	Sunflower		Obtained from IVF, CAAS
Humulus scandens (Lour.) Merr.	Scandent hop		Transplanted from agricultural fields
Impatiens balsamina L.	Balsam		Obtained from IVF, CAAS
Ocimum basilicum L.	Sweet basil		Obtained from IVF, CAAS
Polygonum orientale L.	Smartweed		Obtained from IVF, CAAS
Ricinus communis L.	Castor bean plant	Fengbi 6	Bred by Cash Crop Research Institute, Shanxi Academy of Agricultural Sciences (Taiyuan, China)
Vigna radiata (L.) Wilczek	Mungbean	JILVDOU2	Bred by Baoding Institute of Agricultural Sciences (Baoding, China)
